# Study of the Phase Transitions in the Binary System NPG-TRIS for Thermal Energy Storage Applications

**DOI:** 10.3390/ma13051162

**Published:** 2020-03-05

**Authors:** Sergio Santos-Moreno, Stefania Doppiu, Gabriel A. Lopez, Nevena Marinova, Ángel Serrano, Elena Silveira, Elena Palomo del Barrio

**Affiliations:** 1 Centre for Cooperative Research on Alternative Energies (CIC energiGUNE), Basque Research and Technology Alliance (BRTA), Alava Technology Park, 01510 Vitoria-Gasteiz, Spain; ssantos@cicenergigune.com (S.S.-M.); epalomo@cicenergigune.com (E.P.d.B.); 2 TECNALIA, Basque Research and Technology Alliance (BRTA), Parque Tecnológico de San Sebastián, 20009 Donostia-San Sebastián, Spain; nevena.marinova@tecnalia.com (N.M.); elena.silveira@tecnalia.com (E.S.); 3 Applied Physics II, University of the Basque Country UPV-EHU, 48940 Leioa, Spain; gabrielalejandro.lopez@ehu.eus; 4 Ikerbasque, Basque Foundation for Science, 348013 Bilbao, Spain

**Keywords:** phase change material, thermal energy storage, latent heat storage, neopentyl glycol, TRIS, plastic crystals, globular polyols, subcooling

## Abstract

Neopentylglycol (NPG) and tris(hydroxymethyl)aminomethane (TRIS) are promising phase change materials (PCMs) for thermal energy storage (TES) applications. These molecules undergo reversible solid-solid phase transitions that are characterized by high enthalpy changes. This work investigates the NPG-TRIS binary system as a way to extend the use of both compounds in TES, looking for mixtures that cover transition temperatures different from those of pure compounds. The phase diagram of NPG-TRIS system has been established by thermal analysis. It reveals the existence of two eutectoids and one peritectic invariants, whose main properties as PCMs (transition temperature, enthalpy of phase transition, specific heat and density) have been determined. Of all transitions, only the eutectoid at 392 K shows sufficiently high enthalpy of solid-solid phase transition (150–227 J/g) and transition temperature significantly different from that of the solid-state transitions of pure compounds (NPG: 313 K; TRIS: 407 K). Special attention has also been paid to the analysis of metastability issues that could limit the usability of NPG, TRIS and their mixtures as PCMs. It is proven that the addition of small amounts of expanded graphite microparticles contributes to reduce the subcooling phenomena that characterizes NPG and TRIS and solve the reversibility problems observed in NPG/TRIS mixtures.

## 1. Introduction

Thermal energy storage (TES) is a key element in the energy transformation that our society must undergo in order to alleviate the effects of climate change and the scarcity of fossil resources. Traditionally, TES has been used to improve thermal management and energy efficiency in the sector of heating and cooling in buildings, as well as in industrial heat processes. Nowadays, thermal energy storage might become an essential tool for increasing the use of renewable energies, characterized by their temporal variability. It has experimented a particularly important development coupled to low-medium temperature solar thermal collectors, as well as in concentrating solar thermal power plants. Due to the possibility of storing large amounts of energy at relatively low cost, it has started to be considered as an alternative/complement to the massive storage of energy on the electricity grid, which needs to be provided with greater flexibility to absorb the growing proportion of renewables (photovoltaic (PV), wind) connected to the grid. Also, the recent willingness to decarbonize the building and industrial sectors through electrification opens up new prospects for the use of TES.

Currently, sensible heat storage technologies dominate the market. However, latent heat storage based on phase change materials (PCMs) are particularly attractive technologies for applications where energy has to be stored/delivered over a narrow temperature range or/and when space is a limiting factor. Indeed, PCMs can reversibly absorb or release heat during their phase change processes at nearly constant temperature. Moreover, they enable compact TES systems with volumetric storage capacity five to ten times greater than that of sensible heat storage systems.

The research on PCMs has been increasing tremendously over the last 25 years. Many different types of PCMs have been considered for their use in TES systems [1,2,3]. Over half of the materials studied have transition temperatures below 120 °C, with a large number of possible material categories (i.e., salt hydrates, paraffins, fatty acids, sugar alcohols, etc.). Above 300 °C, only anhydrous salts and metallic alloys have been investigated. At medium temperatures (120–250 °C), which dominates in industrial heat processes, there is a significant drop-off in the number and types of PCMs. Moreover, the vast majority of proposed materials are solid-liquid PCMs with relatively high latent heat of fusion. Nevertheless, the leakage of the liquid phase of these materials at temperatures above their melting point can hinder their application. In that way, prior to their implementation in the final appliance, solid-liquid PCMs are usually confined in a supporting matrix or encapsulated [2], which implies considerable production costs. Solid-solid PCMs can be an attractive alternative to solid-liquid PCMs, avoiding the leakage problem and offering a wide range of possibilities for integration into the TES system, as they can be easily shaped and sized [4,5].

Timmermanns [6] already in 1961 proposed a class of organic molecules referred to as “globular molecules” or “plastic crystals” as solid-solid PCMs. These molecules undergo reversible phase transitions from a low temperature ordered layered structure (tetragonal, orthorhombic, monoclinic etc.) to a high temperature orientationally disordered (FCC or BCC) phase, referred to as “plastic or orientationally disordered crystal (ODIC)”. These polymorphic changes are characterized by high enthalpy of solid-solid phase transition, which results from the reversible breaking of nearest-neighbor hydrogen bonds in the molecular crystals [7]. However, very few alcohol and amine derivatives form these plastic crystals. They are pentaerythritol (PE), pentaglycerin (PG), neopentylglycol (NPG), tris(hydroxymethyl)aminomethane (TRIS) and 2-amino-2-methyl-1,3-propanediol (AMPL), whose crystallographic and thermodynamic properties have been extensively investigated [7,8,9,10,11,12,13,14,15,16,17]. They exhibit solid-solid transition in the range of temperatures from 44 °C (NPG) to 185 °C (PE) with enthalpies of transition from 110 J/g (NPG) to 300 J/g (PE), which make them particularly attractive for TES applications in the industrial sector.

In order to increase the possibilities of application of these materials, the miscibility of their solid phases has also been investigated. The study of the phase diagrams of the binary systems NPG-PG [18,19,20], AMPL-NPG [21], NPG-TRIS [20,22,23], PG-TRIS [18,24,25] and TRIS-AMPL [26] has revealed the existence of invariant reactions (eutectoids, peritectoids) and/or solid-solid solutions over wide concentration ranges, which should allow the use of this class of molecules to store energy at temperatures different from the transition temperatures of pure materials.

This study focusses on NPG-TRIS system. The objective is to assess its usability in TES applications. This involves not only identifying compositions displaying solid-solid transitions at temperatures different from those of NPG and TRIS, but also evaluating their storage capacity. Although previous experimental work carried out by Barrio et al. [22] uses X-ray diffraction and differential scanning calorimetry to establish the phase diagram of NPG-TRIS system, the enthalpy of solid-solid phase transitions has only been reported for pure NPG [7,15,16,17,21] and TRIS [11,15,16,17,18]. The same is true for specific heats and densities [14,21,27,28,29]. Besides, whereas metastable phases are commonly observed in plastic crystals when cooling, this is not a factor that has been considered in their evaluation as TES materials although it can limit their usability. Moreover, there are no previous studies trying to avoid metastability. On the one hand, the present study provides full assessment of NPG-TRIS system for TES applications. On the other hand, it also includes a first attempt to reduce metastability issues by doping the NPG-TRIS mixtures with expanded graphite.

## 2. Experimental

### 2.1. Materials

Neopentylglycol (NPG; C_5_H_12_O_2_) and tris(hydroxymethyl)aminomethane (TRIS; C_4_H_11_NO_3_) with a purity of 99 and 99.8%, respectively, were purchased from Sigma-Aldrich (St. Louis, Missouri, USA). To avoid eventual degradation or hydration, both NPG and TRIS were stored in closed glass containers inside a glove box with an argon atmosphere and levels of oxygen and humidity below 0.1 ppm. Highly conductive expanded graphite powder (SIGRATHERM^®^GFG) was purchased from SGL Carbon SE (Wiesbaden, Germany). The average particle size (D_50_) and powder density are 75 μm and 0.120 g/cm^3^, respectively. The powder’s carbon content is more than 95% and the moisture content less than 5%.

NPG_1−x_TRIS_x_ (0 < x < 1, molar fraction of TRIS) samples were prepared by a simple three-step method consisting in: (1) grinding and mixing of NPG and TRIS in the right proportion; (2) heating up of the sample up to 180 °C (453 K) in hermetically closed glass container (10 mL) to avoid eventual sublimation of NPG; and (3) slow cooling down of the sample followed by annealing at room temperature for at least 1 h in order to avoid freezing of metastable phases commonly observed otherwise. The same procedure was applied to prepare NPG_1−x_TRIS_x_ samples doped with expanded graphite. In all cases, NPG, TRIS and expanded graphite were used as received. Regarding the first step of the preparation method, hand-grinding in a mortar and ball-milling were tested and compared. An 8000 M Mixer/Mill^®^ High-Energy Ball Mill, from SPEX SamplePrep LLC (Metuchen, NJ, USA), was used. The samples (2 g) were grinded in stainless steel vials during 15 min at 875 rpm, employing three stainless steel balls of 1 g each. No differences were observed between the thermal behavior of hand-grinded samples and that of ball-milled ones. Therefore, ball-milling was systematically used due to its efficacy.

### 2.2. Methods

#### 2.2.1. Thermal Analysis and Density

Differential Scanning Calorimetry (DSC) studies were performed with a Q2500 Calorimeter from TA Instruments (New Castle, DE, USA) using T-zero closed aluminum crucibles and samples of about 15 mg. All experiments were performed with argon (50 mL/min) as purge gas.

To determine the phase diagram of NPG-TRIS system, 21 NPG_1−x_TRIS_x_ (0 ≤ x ≤ 1) samples were tested with heating/cooling rate of 1 K/min from 293 K to 453 K. The DSC is calibrated every month for heat flow and temperature using high purity (>99.99%) reference materials indium, tin, zinc and aluminum. In this work, the calibration was checked using indium standard before tests. The thermograms obtained (compensation heat flux vs. temperature) were used to determine phase transition temperatures and corresponding enthalpies of phase transition. In the case of an isothermal phenomenon, the transition temperature is considered to be given by the onset temperature; whereas the shape factor method [30] was used to determine the transition temperature when the phenomenon is no longer isothermal. The enthalpies of phase transition are calculated by integration of the endothermic peaks assuming a linear baseline. The accuracy in the determination of transitions temperatures is about ±1 K, whereas that of the enthalpies of phase transition is ±5%. The same testing conditions were applied to perform the thermal analysis of NPG, TRIS and NPG_1-x_TRIS_x_ samples doped with expanded graphite. Scanning rates between 0.5–10 K/min were employed to study eventual effects of the cooling rate on freezing of metastable phases in NPG, TRIS and NPG_1-x_TRIS_x_ mixtures.

The specific heat of NPG_1-x_TRIS_x_ (0 ≤ x ≤ 1) samples was measured over wide range of temperatures using modulated heating method (accuracy ±5%). The samples were heated by superimposing periodic temperature variations of ±0.2 or ±0.5 K amplitude and 1/60 Hz frequency on a temperature ramp of 1 K/min. The DSC was previously calibrated using sapphire as standard material. 

The true density of NPG_1−x_TRIS_x_ (0 ≤ x ≤ 1) samples was determined by a helium pycnometer (Accupyc II 1340, Micrometrics; Norcross, Georgia, USA) at room temperature.

#### 2.2.2. X-Ray Diffraction (XRD)

X-ray diffraction measurements were performed to verify that during the preparation of NPG_1−x_TRIS_x_ (0 < x <1) samples no degradation or structural changes occur after grinding or heating processes. The diffractograms were obtained with a D8 Discover diffractometer from BRUKER (Billerica, MA, USA) equipped with a LYNXEYE XE detector for ultra-fast diffraction measurements and with the Vario1 monochromator, using a CuKα_1_ radiation with a 1.5419 Å wavelength. Diffractograms have been recorded in the 2*θ* angular range 10–80°, with a step size of 0.02° and a step time of 1.0 s, using a tube voltage of 40 kV and a tube current of 40 mA. All the samples were tested at room temperature.

#### 2.2.3. Liquid-State NMR

The ^1^H-NMR spectra of NPG_1−x_TRIS_x_ (0 < x <1) samples were obtained to determine their composition with high accuracy before and after applying heating treatments. This is especially relevant to check that the composition of the samples does not change during heating because of NPG evaporation. ^1^H-NMR spectra were recorded using a Bruker^®^ 500 Advance III spectrometer (500 MHz for ^1^H and 125 MHz for ^13^C) in the perdeuterated solvent D_2_O in a concentration of ca. 10 mg/mL. The values of chemical shifts (δ) in ppm are referred to tetramethylsilane (Me4Si, TMS) as standard (δ = 0.00 ppm).

## 3. Results and Discussion

### 3.1. Pure NPG and TRIS

**Figure 1:**
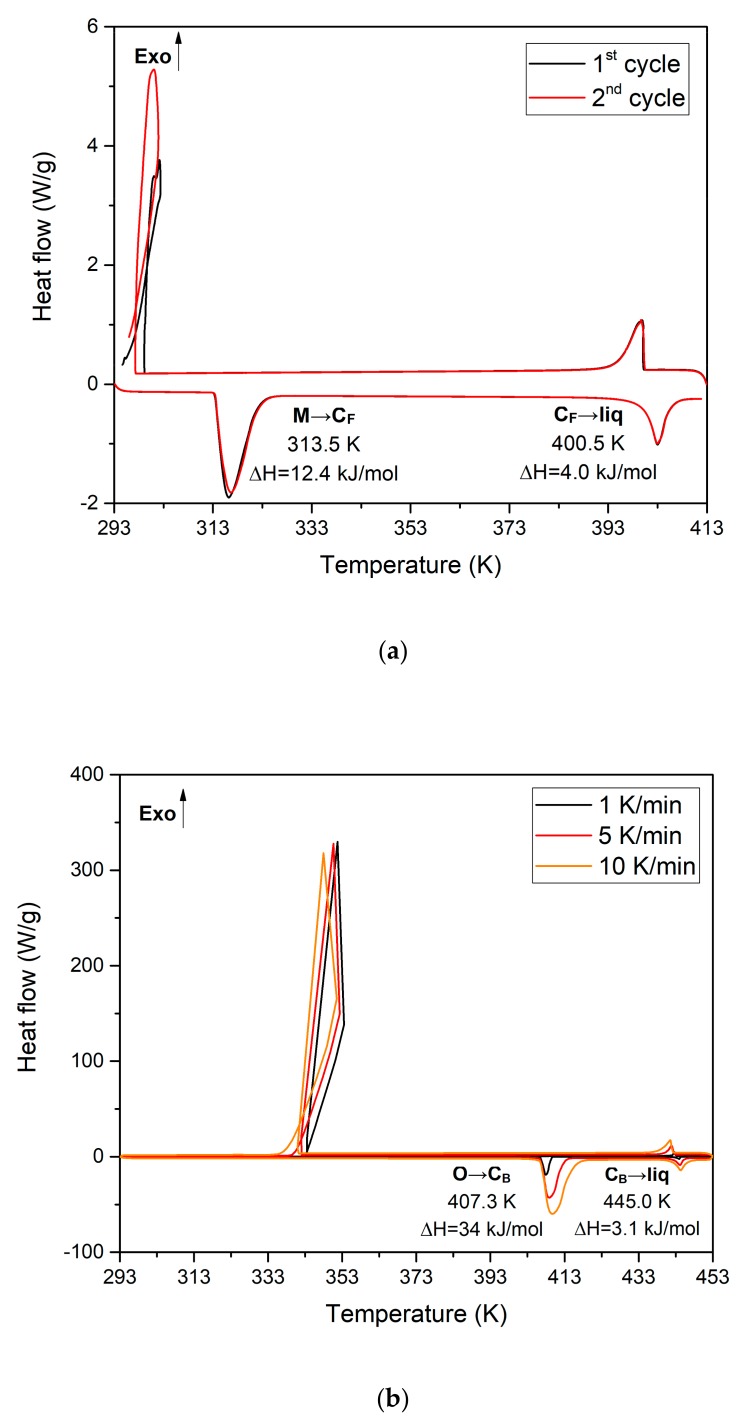
DSC thermograms (compensation heat flux vs. temperature) for (**a**) pure NPG and (**b**) pure TRIS obtained at heating rates of (**a**) 5 K/min for NPG and (**b**) 1, 5 and 10 K/min for TRIS.

NPG and TRIS are tetrahedral molecules derived from neopentane whose crystallographic and thermodynamic parameters have been extensively investigated [11,18,21,22,23]. The crystal structure of the low temperature phase of NPG is monoclinic P2_1_/n, whereas that of TRIS exhibit an orthorhombic lattice with space group Pn2_1_a. High temperature orientationally disordered crystals (ODIC) are face-centered cubic (FCC) and body-centered cubic (BCC) structures for NPG and TRIS, respectively. The results of the thermal analysis carried out in this study are depicted in Figure 1. Two kind of DSC tests have been performed. In the first one, the samples of NPG and TRIS are submitted to three consecutive heating and cooling cycles at a heating/cooling rate of 5 K/min to check the repeatability of the results (Figure 1a). In the second one, the samples are also cycled three times but using different heating/cooling rates (1, 5 and 10 K/min) to analyze the eventual influence of this parameter on metastability (Figure 1b).

For NPG (Figure 1a), the solid-state phase transition from the low temperature monoclinic crystal [M] to the high temperature FCC plastic phase [C_F_] happens at 313.5 K. The high temperature plastic phase [C_F_] melts at 400.5 K. The enthalpy of phase transitions [M] → [C_F_] and [C_F_] → [L] are 119.4 J/g (12.43 kJ/mol) and 37.6 J/g (3.91 kJ/mol), respectively. Figure 1b shows that the low temperature orthorhombic solid phase [O] of TRIS stabilized below 407.3 K, whereas the high temperature BCC phase [C_B_] is stable from 407.3 K to 445 K. The liquid phase [L] appears above 445 K. The enthalpy of the phase transitions [O] → [C_B_] and [C_B_] → [L] are, respectively, 280.7 J/g (33.99 kJ/mol) and 26 J/g (3.15 kJ/mol). As shown in Table 1, these results are in good agreement with those of previous studies.

Moreover, the enthalpy of transition from the low temperature ordered structure to the OCID is almost three times higher for TRIS than for NPG. Indeed, the unusually large enthalpies of these solid-state transformations have been explained in terms of a rotational/vibrational disorder transformation. Benson et al. [14] suggested that hydrogen bonding in polyhydric alcohols held the nearly spherical molecules rigidly in the low temperature crystal phase until, at the transition temperature, all these bonds are broken permitting molecular vibration and rotation. This hydrogen bonding hypothesis was tested by infrared absorption spectroscopy, then further by examining the dependence of the solid-state transition enthalpies (ΔH_TR_) on the number of hydrogen sites able to make hydrogen bonding (acid hydrogens) per molecule (n). The results demonstrated that there is a perfect linear correlation between ΔH_TR_ and n^2^, the transition enthalpy increasing with the number of acid hydrogens per molecule. Accordingly, NPG, with two hydroxyl groups, shows much lower solid-state transition enthalpy than TRIS, that has three hydroxyl groups (-OH) and one amine group (-NH_2_) per molecule.

As shown in Figure 1, whereas undercooling is negligible in the solid-liquid transitions of NPG and TRIS, their solid-solid transitions (NPG: [M] → [C_F_], TRIS: [O] → [C_B_]) display a significant degree of undercooling. Within the range of tested cooling rates (1-10 K/min), the observed undercooling is about 15 K for NPG and 65 K for TRIS. This is typical in polyhydric alcohols and its amine derivatives and could be explained by the degree of disorder and molecular motion in the plastic phase. Indeed, the existence of a high degree of orientational freedom is the most characteristic feature of the plastic crystalline state. According to Rao [31], among possible rotational motions in crystals (free rotation, rotational diffusion and jump reorientation), collision-interrupted molecular rotation is the most likely one in plastic crystals. Preferential orientations of tetrahedral, or neopentane-like, molecules have been studied by Guthrie et al. [32] from steric and symmetry considerations. The results of this theoretical study were later confirmed by molecular dynamic simulations [33] and configurational entropy calculations [34]. According to Guthrie’s work, the molecules of NPG display one single configuration in the low temperature ordered crystal while they exhibit 60 configurations (10 molecular orientations that each possesses six possible hydroxymethyl conformations) in the plastic phase. Therefore, below but close to the solid-state transition temperature, the probability that a particular hydroxyl group have a juxtaposed hydroxyl group from a nearest neighbor molecule with which to form a hydrogen bond is very low (p = 1/60 × 1/60 < 0.028%). By further reducing the temperature, the reduction in volume experienced by the plastic phase should likely reduce the number of preferential molecular orientations and thus facilitate the appearance of the stable crystalline phase. This interpretation of undercooling in plastic crystals is quite speculative and further research will be needed to achieve a well-established theory to explain this phenomenon.

**Figure 2:**
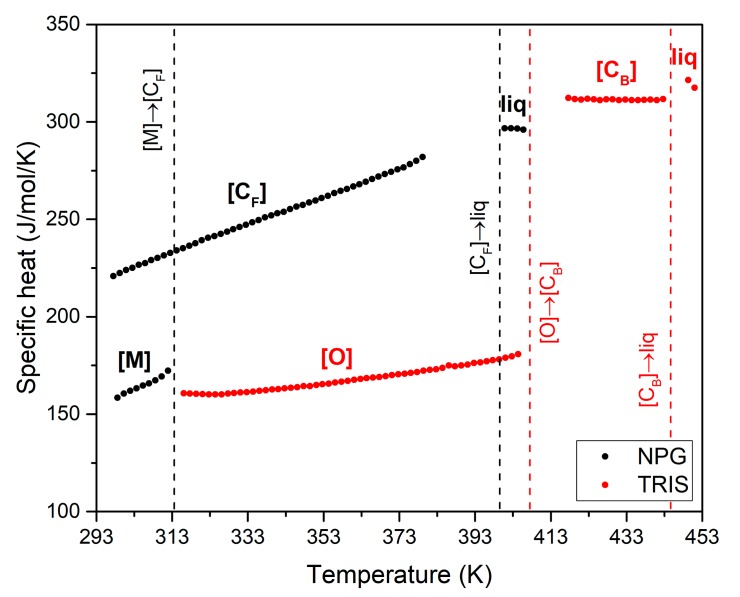
Measured values of specific heat for pure NPG (black) and TRIS (red).

Another measured thermal property of NPG and TRIS is the specific heat. The results depicted in Figure 2 show that the higher the phase disorder, the higher the specific heat. The highest values correspond to the liquid phases, while the lowest are those of the ordered monoclinic (NPG) and orthorhombic (TRIS) crystal structures. In the scanned temperature range, the specific heat of the solid phases of NPG increases linearly with temperature. The same applies for the orthorhombic crystal structure of TRIS, while the specific heat values in the plastic phase are practically insensitive to temperature.

Table 2 summarized the results obtained at temperatures characteristic of the low temperature ordered phase, the plastic phase and the liquid. The results in the table show that there is a quite good agreement between the values of specific heat measured for NPG in this study and those reported in the literature [28]. However, the values for TRIS are 15–20% lower than those measured by Suresh et al. [29].

### 3.2. Phase Diagram of NPG-TRIS System

The phase diagram of NPG-TRIS binary system was established for the first time by Barrio et al. [22]. Using both crystallographic and thermal analysis, they identified two eutectoid invariants and one peritectic invariant. The first eutectoid ([M + O + C_F_]) was reported at 310 ± 1 K with composition of 3.5 mol% of TRIS, the second one ([O + C_F_ + C_B_]) was observed at 392.5 ± 1 K and 57 mol% of TRIS, and the peritectic invariant ([C_F_ + C_B_ + L]) was identified at 410.7 ± 2 K with peritectic composition of 48.4 mol% of TRIS. Based on the experimental results of Barrio et al. [22], the NPG-TRIS binary system was calculated by Shi et al. [20] using regular and sub-regular solution models and CALPHAD method and proven to be in very good agreement with experimental data.

In this study, the NPG-TRIS system has been investigated by thermal analysis. Twenty-one different compositions have been studied. Compared to the previous work of Barrio et al. [22], a finer exploration of the peritectic region has been carried out including 11 compositions within the composition range from x = 0.45 to x = 0.55. The DSC tests were performed by heating the samples from 20 °C (most of them) or 100 °C (those used to refine the peritectic plateau) up to 180 °C at a heating rate of 1 K/min. XRD and liquid NMR were used to check that neither samples preparation nor their thermal treatment leads to degradation, unexpected structural changes or compositional changes due to NPG sublimation (Appendix A).

**Figure 3:**
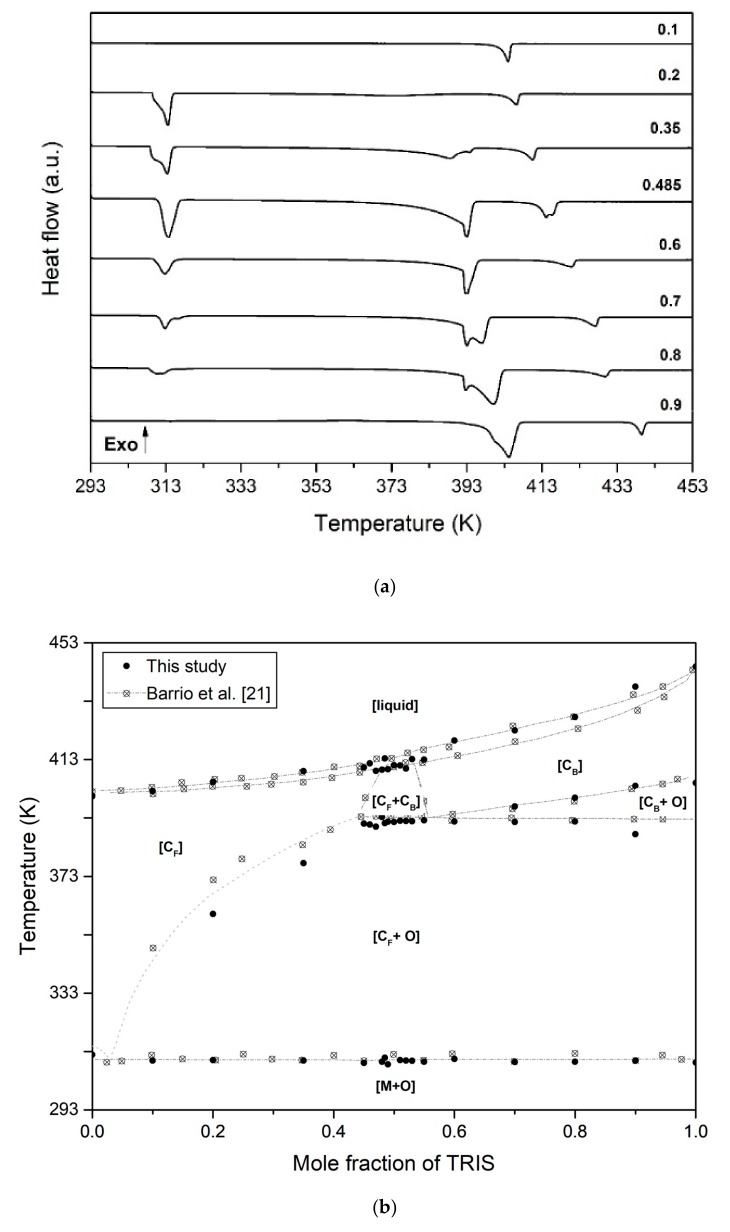
(**a**) DSC thermograms for eight selected compositions and (**b**) phase diagram of the NPG-TRIS system proposed by Barrio et al. [22] (discontinuous lines), with the experimental points of these authors and those obtained in this study.

The results of the thermal analysis carried out are summarized in Table 3 and Figure 3. Table 3 shows the temperature values of the phase transitions observed for each of the 21 tested compositions. The DSC thermograms for 8 selected compositions are depicted in Figure 3a. Finally, Figure 3b displays the phase diagram of NPG-TRIS system proposed by Barrio et al. [22] together with the experimental points obtained in this study.

As shown in Figure 3a (see also Table 3) the calorimeter signal of all the samples (with the exception of pure compounds) shows an endothermic effect at 310 ± 1.0 K, that corresponds to the eutectoid [M + O + C_F_] which extends over the whole range of studied compositions. For the samples with TRIS mole fraction beyond 0.45 (with the exception of pure TRIS), a second endothermic signal is observed at 392 ± 2.0 K, corresponding to the second eutectoid [M + O + C_B_]. The peritectic plateau is evidenced by a third endothermic effect observed at 410 ± 1.0 K in the samples with TRIS mole fraction between 0.45 and 0.53. The last endothermic peak in each composition corresponds to the melting.

Apart from already mentioned isothermal transitions, the calorimeter signal for samples with TRIS mole fraction below 0.45 show a progressive endothermic effect just after the low temperature eutectoid (Figure 3a), that results from the slow diffusion of TRIS molecules toward the cubic lattice C_F_ [22]. As shown in Figure 3b, the temperature-composition dependence of the boundary solid solution C_F_ between the two eutectoids (first superior solvus line) ranges from 310 K to 392 K. Similarly, a progressive endothermic effect is observed just after the high temperature eutectoid in the samples with TRIS mole fraction beyond 0.55 (with the exception of pure TRIS). This is due to the progressive transformation of orthorhombic crystal structure of TRIS molecules into BCC disordered phase. As it can be seen in Figure 3b and Table 3, the second superior solvus line ranges from 392 K to 403 K.

**Figure 4:**
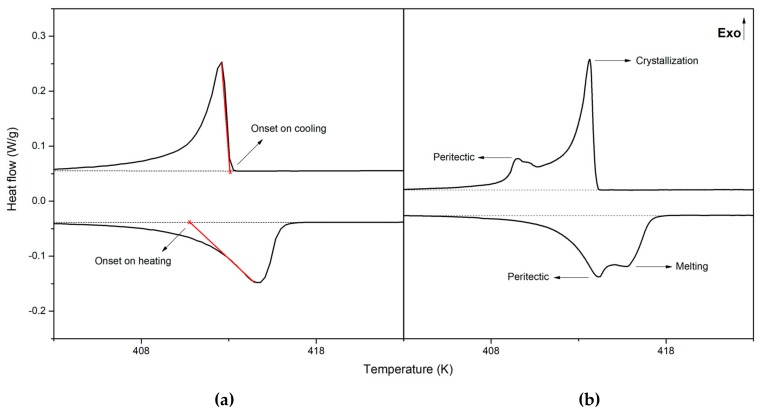
(**a**) Zoom of the DSC thermogram obtained for NPG-TRIS mixture with 0.7 mole fraction of TRIS showing the melting/crystallization process; (**b**) Zoom of the DSC thermogram obtained for NPG-TRIS mixture with 0.485 mole fraction of TRIS showing the peritectic transition and melting/crystallization process.

In summary, it is worth to note that there is a very good agreement between the results obtained in this study and those reported by Barrio et al. [22]. The main difference appears in the first superior solvus (Figure 3b), which shows lower temperature values in this study but better agreement with the computational model by Shi et al. [20]. Some differences can also be appreciated in the transition temperatures associated to the melting processes. Barrio et al. [22] propose an isomorphous phase diagram in which solid phases [C_F_] and [C_B_] are completely miscible, thus forming a continuous solution. Therefore, they give temperature values for both the solidus and the liquidus lines. However, the DSC thermograms obtained in this study do not allow to make a clear distinction between both lines so that only the onset temperature of the endothermic peaks of melting is given. As shown in Figure 4a, the shape of the melting/crystallization peaks obtained in this study does not differ from that typically observed in melting/crystallization processes without solid solutions. Nevertheless, it is remarked that the starting crystallization temperature is always higher than the onset melting temperature, which would support the hypothesis of Barrio et al. [22] about the miscibility of [C_F_] and [C_B_] phases. The similarity between the crystalline structures of both phases would also support this hypothesis. In addition, for peritectic transformation to occur, there must be a region where phases [C_B_] and [L] (cf. [C_F_] and [L]) coexist. Consequently, the apparent differences between our results and those of Barrio et al. [22] do not reflect any fundamental contradiction, but only different ways of exploiting the DSC thermographs.

### 3.3. Enthalpies of Transition and Specific Heats

The enthalpy changes associated to the different transitions observed in NPG-TRIS mixtures have been determined from DSC thermograms and are analyzed here. We focus on isothermal or quasi-isothermal transformations, which are those of interest for the storage of thermal energy by latent heat.

In the second column of Table 4 the enthalpy of transition from the ordered crystal structure to the plastic phase of NPG and TRIS is reported. The values of enthalpy associated to the low temperature eutectoid reaction ([M] + [O] → [C_F_] at 310 ± 1.0 K) are given in the third column, whereas those related to the high temperature eutectoid are in the next column. For compositions 0.45 < x < 0.55, the values reported correspond to the isothermal transition [C_F_] + [O] → [C_F_] + [C_B_] at 392 ± 2.0 K; while at compositions beyond x = 0.55, they correspond to the whole transition [C_F_] + [O] → [C_B_] which take place between the eutectoid plateau (392 ± 2.0 K) and corresponding superior solvus (from 392 K up to 403 K depending on the composition). The enthalpy of melting or the sum of the enthalpies of melting and peritectic transition is given in the last column. It must be noticed that within the peritectic region, the liquidus line and the peritectic plateau are very close in temperature, so as the peritectic reaction and the melting appear as overlapping endothermic peaks in the DSC thermograms (see Figure 4b). This is the reason why the corresponding enthalpies of transition are not given separately but as a sum.

**Figure 5:**
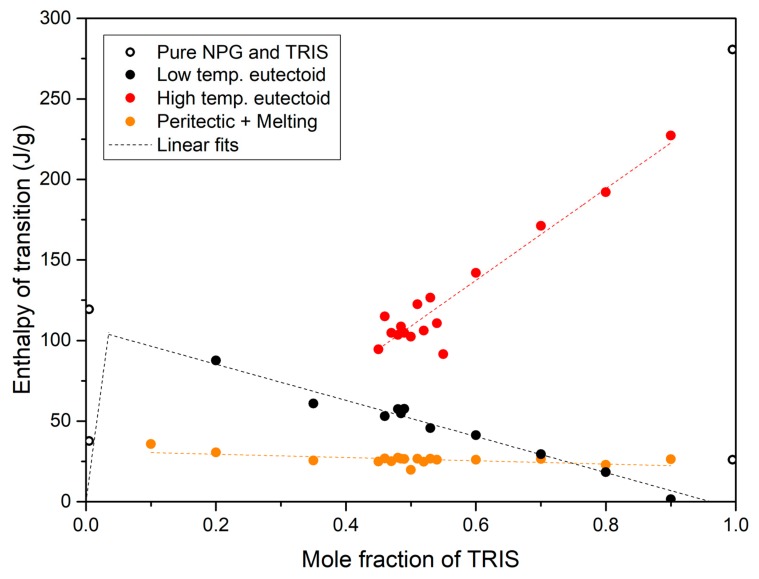
Enthalpy changes related to the different transitions in the NPG-TRIS system: experimental data (symbols) and calculated linear trends (discontinuous lines).

The experimental values of enthalpy in Table 4 are depicted in Figure 5 against the mole fraction of TRIS. The discontinuous lines are calculated linear trends. In the case of the low temperature eutectoid transition, the regression line calculated on the experimental data (symbols) has been used, together with the eutectoid [M + O + C_F_] composition of 0.035 mole fraction of TRIS proposed by Barrio et al. [22], to determine the so called Tammann plot (dependence of enthalpy related to the eutectoid effect on molar fraction). We remind that as a consequence of the lever rule, the enthalpy of transition should descend linearly on either side of the eutectoid point. The maximum value appears at the eutectoid point (0.035 mole fraction of TRIS) and, by extrapolating the regression line, is estimated to be about 105 J/g.

A shown in Figure 5, the enthalpy of melting (including the enthalpy of the peritectic transition when applies) is quite low: it ranges from 37 J/g to 18 J/g, depending on the composition. This is a typical feature of plastic crystals. Indeed, although the plastic phase has a cubic crystal structure with the center of mass of the molecules at fixed positions (FCC for NPG, BCC for TRIS), it is characterized by highly rotationally and vibrational disorder, so the entropy change when melting is relatively low. Therefore, exploiting either melting or peritectic transition or both transformations together in storage applications makes no much sense.

Higher storage energy capacity can be obtained through the low temperature eutectoid transition. As it can be seen in Figure 5, the enthalpy of this solid-solid transition shows a maximum value of 105 J/g for 0.035 mole fraction of TRIS (eutectoid point [M + O + C_F_]), which is quite high. However, using this transition for TES has no clear advantages compared to pure NPG. Indeed, the eutectoid transition takes place at 310 K, only 3 K approx. below the solid-solid transition of pure NPG, and the enthalpy of transition is 14 J/g lower (105 J/g vs. 119 J/g). It should also be noted that the enthalpy of transition at the eutectoid point is lower (10 J/g approx.) than that obtained by multiplying the [M] phase initial content in the mixture by the enthalpy of the eutectoid reaction [M] + [O] → [C_F_] (119 J/g). This comes from the fact that NPG and TRIS have different number of hydroxyl groups. As a consequence, the structure of hydrogen bridges in the low temperature crystal structure of NPG changes when TRIS molecules are incorporated, so as some -OH or -NH_2_ not form hydrogen bonds [17].

The high temperature eutectoid transition has greater interest. Indeed, the enthalpy associated to this transition ranges from 95 J/g (at 0.45 mole fraction of TRIS) up to 245 J/g (close to pure TRIS). Again, the observed enthalpy changes are lower than that of pure TRIS solid-solid transition. They are even below the enthalpy values corresponding to the solid-solid transition of the fraction of TRIS in the mixture. As already explained, this reflects the effect of incorporating NPG molecules in the low temperature crystal structure of TRIS, which makes the number and energy of hydrogen bonds to be reduced. In spite of it all, high enough enthalpy values (>150 J/g), comparable to those of paraffin waxes and salt hydrates in the same range of temperature, can be obtained by using NPG-TRIS mixtures with composition ≥0.6 mole fraction of TRIS. Moreover, the whole transition [C_F_ + O] → [C_B_] takes place in a narrow temperature range at 39-53 K below the solid-solid phase transition of pure TRIS (Figure 3b).

**Figure 6:**
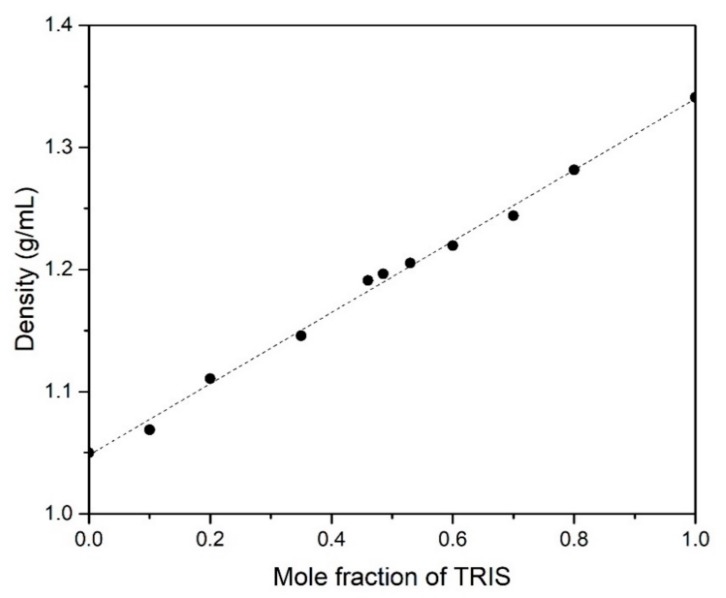
Measured values of true density (symbols) and calculated linear trend (discontinuous line).

In practice, not only the gravimetric energy density is important, but also the volumetric energy density, which determines the volume of the storage system. Figure 6 shows the true density values of different NPG-TRIS mixtures measured at room temperature.

As expected by the general rule of mixing, the density value increases linearly with the TRIS content in the mixtures, from 1.05 g/mL (NPG) to 1.34 g/mL (TRIS) at a rate of 0.293 g/mL per mole fraction unit of TRIS. The estimated values of volumetric energy density (enthalpy of phase transitions in kWh/m^3^) from measured values of enthalpies of transition and densities are reported in Table 5.

**Figure 7:**
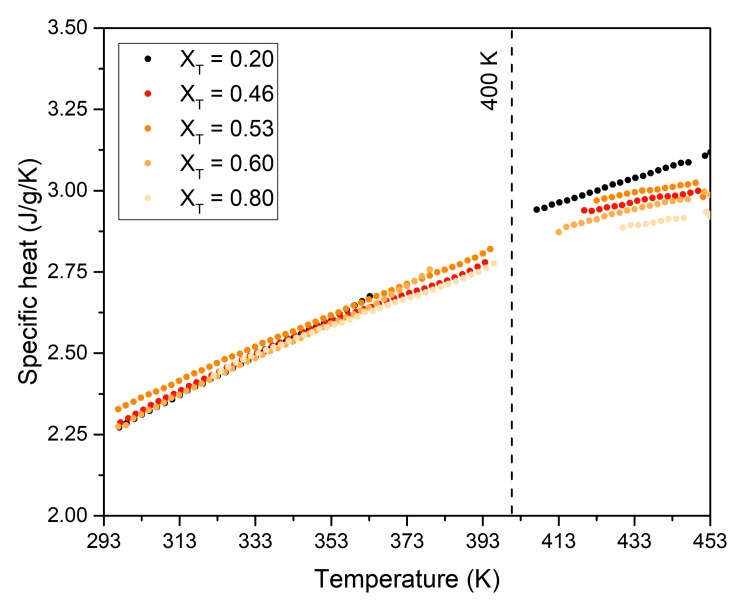
Specific heat as function of temperature for the liquid phase of different NPG-TRIS mixture (0.2 to 0.8 mole fraction of TRIS). The data at temperatures above 400 K represent the specific heat of stable liquid phase, whereas those below 400 K correspond to metastable liquid phase achieved during cooling.

Although specific heat is a secondary parameter in thermal storage by isothermal or quasi-isothermal phase change phenomena, we have also analyzed it. Measured data of specific heat in liquid phase, including stable and metastable liquid phases, are depicted in Figure 7. As expected, the specific heat increases with temperature for all compositions. Moreover, for a given temperature, the specific heat tends to slightly decrease when the content of TRIS in the mixture increases.

**Figure 8:**
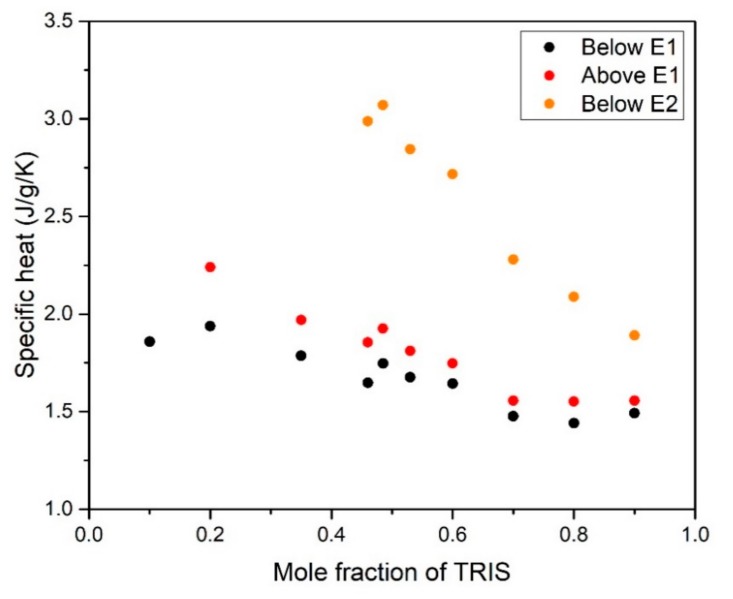
The specific heat values obtained just below (303–307 K) and above (317–321 K) the low temperature eutectoid (E1), as well as that close below (362–363 K) the high temperature eutectoid (E2).

The measurements in the solid phases are more difficult because of the different phase transitions taking place, whose endothermic effects interfere with the calorimeter signal of the specific heat. The specific heat values obtained just below and above the low temperature eutectoid transition (current phases: [M] + [O]), as well as that close below the high temperature eutectoid (current phases: [C_F_] + [O]), are depicted in Figure 8. 

The figure shows that, in all cases, the specific heat decreases increasing the TRIS content in the mixture. This behavior was expected considering that the orthorhombic ([O]) crystal structure of TRIS has lower specific heat than the monoclinic ([M]) and plastic phase ([C_F_]) of NPG (see Figure 2).

### 3.4. Metastability Issues

We have already identified undercooling as a factor limiting the usability of the solid-solid transitions taking place in pure NPG ([M] → [C_F_]) and pure TRIS ([O] → [C_B_]) in thermal energy storage applications (Section 3.1). Additional metastability issues affecting the phase transitions in NPG-TRIS system are here discussed. To illustrate them, let us consider NPG-TRIS mixture with 0.485 mole fraction of TRIS, which exhibit all possible transitions taking place in NPG-TRIS system (except [M] → [C_F_] and [O] → [C_B_]) as well as all metastability problems observed in other compositions.

**Figure 9:**
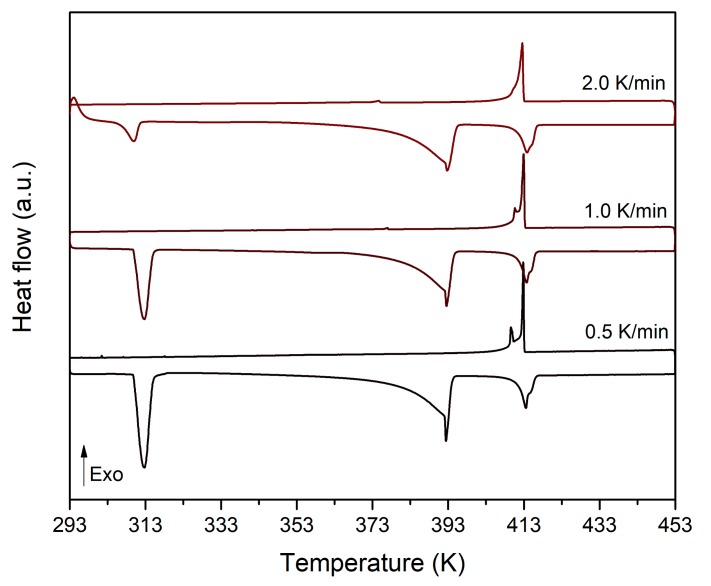
DSC thermograms obtained for different NPG_0.515_TRIS_0.485_ samples submitted to different heating/cooling rates (0.5 to 2 K/min) between 293 K and 453 K.

Figure 9 represents the DSC thermograms obtained for different NPG_0.515_TRIS_0.485_ samples submitted to different heating/cooling rates (0.5 to 2 K/min) between 293 K and 453 K. At the lowest heating rate (0.5 K/min), the DSC thermogram shows four endothermic peaks that correspond to the following transitions (see Figure 4): [M + O] → [C_F_ + O] at 310 K (low temperature eutectoid), [C_F_ + O] → [C_F_ + C_B_] at 392 K (high temperature eutectoid) and [C_F_ + C_B_] → [C_B_ + L] (peritectic) followed by [C_B_ + L] → [L] (melting) at 410 K. However, on cooling, only two exothermic peaks appear in the thermogram. They correspond to the phase transitions [L] → [C_B_ + L] and [C_B_ + L] → [C_F_ + C_B_], which happen with negligible undercooling. The high temperature eutectoid reaction [C_F_ + C_B_] → [C_F_ + O] does not take place and, therefore, the low temperature eutectoid transition [C_F_ + O] → [M + O] neither. A metastable state in which [C_F_] and [C_B_] phases coexist is reached at the end of the cooling stage. As the heating/cooling rate increases, not only does this metastability persist, but there is also a progressive decrease of the endothermic peaks corresponding to the low temperature eutectoid transition. The enthalpies of phase transition measured over heating are reported in Table 6. It can be seen that the enthalpies of the high temperature eutectoid reaction and that of the peritectic transition and melting are not affected by the heating rate, whereas the enthalpy of the low temperature eutectoid reaction is significantly reduced for heating rates beyond 1 K/min. This is likely due to the slow diffusion of NPG molecules toward the cubic lattice C_F_ [22].

**Figure 10:**
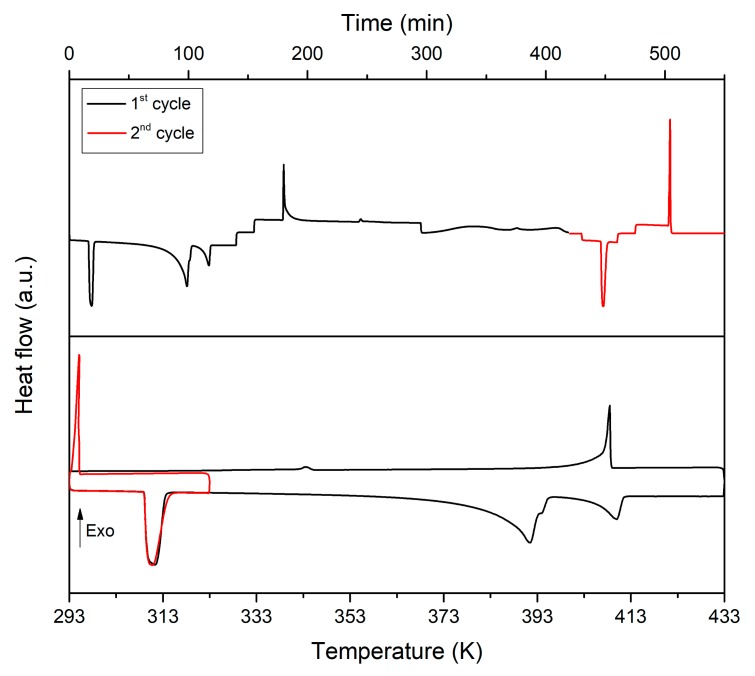
DSC results obtained for the NPG-TRIS sample with composition equal to 0.485 mole fraction of TRIS with heating/cooling rate of 1 K/min and annealing for 1 h at 293 K between the first and second cycles.

Let us come back to the phase transitions [C_F_ + C_B_] → [C_F_ + O] and [C_F_ + O] → [M + O]. To investigate which, or if both, is responsible for the observed metastability, a new DSC experiment was carried out on a NPG_0.515_TRIS_0.485_ sample. The sample was first submitted to heating and cooling between 293 K and 433 K with a heating/cooling rate of 1 K/min. After the cooling stage, the sample was kept at 293 K for 1h before subjecting it to a second heating and cooling cycle between 293 K and 323 K at a rate of 1 K/min. The calorimetric signals corresponding to this experiment are depicted in Figure 10. As expected, the phase transitions [C_F_ + C_B_] → [C_F_ + O] and [C_F_ + O] → [M + O] are not observed during the cooling step of the first DSC cycle. However, during annealing (1 h isotherm at 293 K-Times 300–400 min in Figure 10), a progressive weak exothermic effect can be appreciated corresponding to the transition from metastable phase [C_F_ + C_B_] to stable [M + O]. This is confirmed by the appearance of the endothermic peak associated to the low temperature eutectoid during the second DSC cycle. The latter also shows that the phase transition [C_F_ + O] → [M + O] takes place over cooling, although it shows a significant degree of undercooling (c.a. 12 K). All this indicates that the apparent lack of reversibility in the transition from [C_F_ + C_B_] to [M + O] is mainly determined by slow kinetic of the phase transition [C_F_ + C_B_] → [C_F_+O].

**Figure 11:**
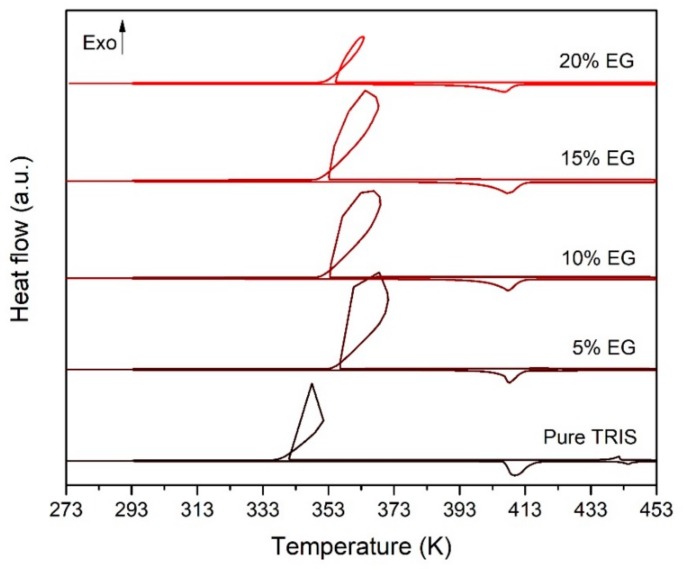
DSC thermograms obtained at a heating/cooling rate of 1 K/min for TRIS samples doped with EG micro-particles. EG content: 0%wt, 5%wt, 10%wt, 15%wt and 20%wt.

As a first attempt to solve metastability issues, pure NPG and TRIS as well as NPG - TRIS mixtures have been doped with expanded graphite (EG) microparticles (average particle size = 75 μm). Figure 11 shows the DSC thermograms obtained for pure TRIS doped with different EG weight percentages. It can be seen that the addition of EG micro-particles has a beneficial effect on undercooling, although still insufficient for the use of TRIS as phase change material in thermal energy storage applications. Similar results were obtained for pure NPG doped with EG microparticles.

Table 7 summarizes the observed effects of added microparticles on undercooling as well as on the temperature and enthalpy of solid-solid transition.

Table 7 shows that the undercooling of pure TRIS is progressively reduced when increasing the EG content, from 65 K for pure TRIS to 41.6 K for TRIS doped with 20%wt of EG. That is, the percentual reduction of undercooling goes from 15% to 36% approx. In the case of NPG, EG particles yield to an undercooling reduction of about 5 K (35% reduction) for all tested composites. It can also be observed that EG microparticles have significant effect on the temperature and enthalpy of the transition from the orthorhombic crystal structure of TRIS to its plastic phase. The transition temperature (T_TR_) is moderately lowered as the EG content is increased, whereas the enthalpy (ΔH_TR_) is abnormally reduced. Indeed, measured values of ΔH_TR_ are 30–50 J/g approx. less than the enthalpy change calculated by applying the rule of mixtures (ΔH_TR_ (cal.) = (1 − weight fraction of EG) × ΔH_TR_). These effects are progressively amplified by increasing the amount of added EG, and, to a lesser extent, they can also be appreciated in NPG. The reduction of T_TR_ and ΔH_TR_ likely indicates that EG microparticles modify the hydrogen bonds structure of the low temperature crystal structure of TRIS and NPG. Added microparticles probably act as structural defaults that reduce the average number of hydrogen bonds by molecule. This leads to lower enthalpy of the solid-solid transition toward the plastic phase, on the one side; and facilitates the transition from the plastic phase to the ordered crystal structure, on the other side.

**Figure 12:**
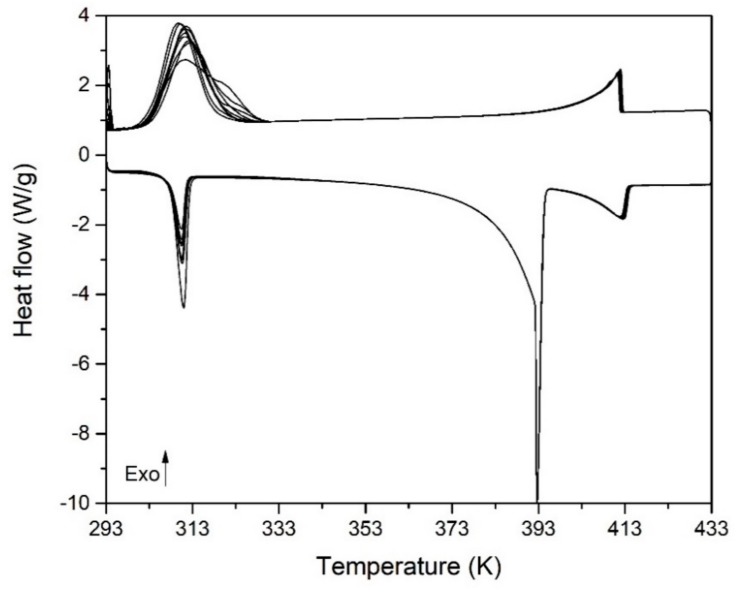
DSC thermograms obtained at heating/cooling rate of 1 K/min for NPG_0.515_TRIS_0.485_ mixture with 10%wt. of EG.

The effect of EG microparticles in the phase transitions [C_F_ + C_B_] → [C_F_ + O] and [C_F_ + O] → [M + O] has also been investigated. Figure 12 shows the DSC thermogram obtained for a NPG_0.515_TRIS_0.485_ sample doped with 10%wt of EG microparticles. The figure shows that, contrary to the case of undoped mixture, the phase transitions [C_F_ + C_B_] → [C_F_ + O] and [C_F_ + O] → [M + O] take place within the scanned temperature range, although they display high undercooling degree which makes them of few interest for TES applications.

## 4. Conclusions

The polyhydric alcohols neopentylglycol (NPG) and tris(hydroxymethyl)aminomethane (TRIS) exhibit solid state transformations in which hydrogen bonds between molecules are broken and rotational and vibrational disorder is introduced. These transitions from an ordered crystal structure to a disordered one are characterized by high enthalpy changes, which makes NPG and TRIS very attractive phase change materials for thermal energy storage applications. NPG enables energy storage around 313 K with gravimetric energy density of 119 J/g, whereas TRIS stores heat at 407 K with 281 J/g storage capacity. NPG-TRIS binary system has been investigated in this study looking for mixtures that cover transition temperatures different from those of pure compounds, thus extending their potential use in TES applications. The phase diagram of NPG-TRIS system has been studied by thermal analysis. The results show that NPG-TRIS mixtures exhibit similar solid-state transformations than pure compounds, apparently following the same mechanisms. The phase diagram presents three invariants (two eutectoids and one peritectic) that could be used to store heat under isothermal or near-isothermal conditions. However, only the eutectoid at 392 K shows sufficiently high enthalpy of solid-solid phase transition and transition temperature significantly different from that of the solid-state transitions of pure compounds. This eutectoid extends over wide range of compositions (mole fraction of TRIS above 0.45) providing increasing energy storage capacity values as the TRIS content increases, from 95 J/g (0.45 mole fraction of TRIS) up to 245 J/g (close to pure TRIS).

In spite of numerous assets of NPG and TRIS, undercooling has been identified as a limiting factor for their use in TES applications. The observed undercooling degree is about 15 K for NPG and 65 K for TRIS. This is typical in polyhydric alcohols and its amine derivatives and could be explained by the existence of a high degree of orientational freedom in their plastic phase, which leads to very low probability that hydroxyl groups from two neighbor molecules juxtapose and form a hydrogen bond. It has been proven that adding expanded graphite (EG) microparticles to NPG and TRIS has a beneficial effect on undercooling, which is significantly reduced. For NPG, the reduction is of about 5 K regardless of the EG content. In the case of TRIS, the higher the EG content, the greater the reduction the undercooling (65 K of TRIS to 41.6 K of TRIS doped with 20%wt of EG). In both cases, this effect on undercooling is accompanied by abnormal reduction of the enthalpy of the solid-state transition, which indicates that EG microparticles modify the hydrogen bonds structure of the ordered crystal structure of TRIS and NPG, so as some -OH or -NH_2_ not form hydrogen bonds. The resulting reduction of the average number of hydrogen bonds per molecule should facilitate the transition from the plastic phase to the ordered crystal structure and could explain the undercooling mitigation observed by adding EG particles.

NPG-TRIS mixtures not only have subcooling problems, but also certain solid-state transformations show slow kinetics. In particular, the transitions [C_B_] → [C_F_ + O], [C_F_ + C_B_] → [C_F_ + O] and [C_F_] → [C_F_ + O] exhibit huge undercooling and need long annealing times to be completed. It has been proved that the addition of EG microparticles (10%wt) has not only significant effect on undercooling, which is strongly reduced, but also significantly improve the kinetics of these transitions.

Although the addition of EG microparticles improves the behavior of NPG, TRIS and their mixtures, the reduction of subcooling achieved is still insufficient for the use of these materials in thermal storage applications. A better understanding of the mechanisms behind the phenomenon of undercooling in plastic crystals will be needed to guide the search for more efficient doping nanoparticles.

## Figures and Tables

**Table 1:** Crystal structures, temperature and enthalpy of transition from the low temperature ordered structure to the high temperature ODIC (T_TR_, ΔH_TR_), and melting point and latent heat of fusion of the ODIC (T_m_, ΔH_m_) for pure NPG and TRIS.

Compound	Low Temp. Phase	T_TR_ (K)	ΔH_TR_ (kJ/mol)	High Temp. Phase	T_m_ (K)	ΔH_m_ (kJ/mol)	Ref.
NPG	Monoclinic	315.0	13.6	FCC	399.0	4.6	[23]
		315.0	12.1		403.2	4.4	[21]
		314.6	12.8		401.3	4.4	[22]
		313.5	12.4		400.5	4.0	This study
TRIS	Orthorhombic	408.0	32.7	BCC	445.0	3.3	[23]
		407.3	32.9		446.0	3.0	[11]
		406.8	34.0		442.7	3.7	[18]
		407.3	34.0		445.0	3.1	This study

**Table 2:** Specific heat value (C_p_) for temperatures characteristic of the low temperature ordered phase, the plastic phase and the liquid of NPG and TRIS.

Compound	Crystal Structure	T (K)	C_p_ (J/mol/K) This Study	C_p_ (J/mol/K) Refs. [28,29]
NPG	Monoclinic crystal structure	310	169.3	178.3
	FCC plastic phase	379	281.9	285.0
	Liquid	401	296.6	313.3
TRIS	Orthorhombic crystal structure	402	179.6	230.0
	BCC plastic phase	421	311.4	363.3
	Liquid	450	319.1	396.6

**Table 3:** Characteristic temperatures for the different phase transitions in the NPG-TRIS system: T_e1_, eutectoid invariant [M + O + C_F_]; T_sv1_, first superior solvus corresponding to the change [O + C_F_] to [C_F_]; T_e2_, eutectoid invariant [O + C_F_ + C_B_]; T_sv2_, second superior solvus corresponding to the change [O + C_B_] to [C_B_]; T_p_, peritectic invariant [C_F_ + C_B_ + L]; T_liq_, liquidus.

Mole Fraction of TRIS	T_e1_ (K)	T_sv1_ (K)	T_e2_ (K)	T_sv2_ (K)	T_p_ (K)	T_liq_ (K)
0 (NPG)	(313.5) ^1^					400.8
0.10	310.5					402.2
0.20	310.5	356.8				403.4
0.35	310.2	378.1				407.2
0.45	n.e ^2^		389.2			410.5
0.46	309.0		391.4		412.1	414.1
0.47	n.e ^2^		390.5		409.2	413.6
0.48	309.6		393.5		409.6	413.2
0.485	311.7		391.8		413.1	413.1
0.49	308.7		394.9		409.8	413.4
0.50	n.e ^2^		391.8		411.1	414.7
0.51	n.e ^2^		392.2		411.1	413.4
0.52	n.e ^2^		392.1		410.1	414.6
0.53	311.5		392.0			413.2
0.54	n.e ^2^		391.8			412.8
0.55	n.e ^2^		392.3	392.3		413.1
0.60	310.4		392.1	392.1		415.3
0.70	309.6		391.9	395.7		423.0
0.80	309.2		392.0	398.3		424.2
0.90	309.3		397.6	402.7		437.7
1 (TRIS)	(407.3) ^1^					445.0

^1^ The values in parentheses are out of the invariants. ^2^ n.e = not explored, the samples were heated from 100 °C up to 180 °C.

**Table 4:** Enthalpies of phase transition in NPG-TRIS system: ΔH_pure_, pure NGP and TRIS, ΔH_e1_, low temperature eutectoid reaction; ΔH_e2_, high temperature eutectoid transformation; ΔH_p+melting_, peritectic reaction (when applies) and melting.

Mole Fraction of TRIS	ΔH_pure_	ΔH_e1_	ΔH_e2_	ΔH_p+melting_
	J/g; (J/cm^3^)	J/g; (J/cm^3^)	J/g; (J/cm^3^)	J/g; (J/cm^3^)
0 (NPG)	119.4 (125.4)	-	-	37.6 (39.5)
0.1	-		-	35.7 (38.2)
0.2	-	87.6 (97.3)	-	30.6 (34.0)
0.35	-	60.9 (69.8)	-	25.6 (29.3)
0.45	-	n.e ^1^	94.5	25
0.46	-	53.0 (63.1)	114.9 (163.8)	26.8 (31.9)
0.47	-	n.e ^1^	104.8	25.1
0.48	-	57.4	103.5	27.3
0.485	-	54.8 (65.6)	108.6 (129.9)	26.6 (31.8)
0.49	-	57.6	104.7	26.5
0.5	-	n.e ^1^	102.3	19.7
0.51	-	n.e ^1^	122.4	26.7
0.52	-	n.e ^1^	106.2	24.8
0.53	-	45.7 (55.1)	126.5 (152.5)	26.7 (32.2)
0.54	-	n.e ^1^	110.7	26
0.55	-	n.e ^2^	91.6	14.6
0.6	-	41.3 (50.4)	141.9 (173.1)	26 (31.7)
0.7	-	29.5 (36.7)	171.1 (212.8)	26.5 (33.0)
0.8	-	18.4 (23.6)	192 (246.1)	22.9 (29.3)
0.9	-	1.5	227.2	26.4
1 (TRIS)	280.7 (376.4)	-	-	26 (34.9)

^1^ n.e = not explored, the samples were heated from 100 °C up to 180 °C.

**Table 5:** Estimated volumetric energy density associated to the different phase transitions in NPG-TRIS system.

Phase Transition	Volumetric Energy Density (kWh/m^3^)
NPG solid-solid transition [M] → [C_F_]	34.7
Low temperature eutectoid transition	0–38.8 depending on the composition
High temperature eutectoid transition	31–91.3 depending on the composition
Melting (including peritectic transition)	11–9.6 depending on the composition
TRIS solid-solid transition [O] → [C_B_]	104.5

**Table 6:** Enthalpy of phase transition (J/g) associated to the endothermic phenomena appearing in the DSC thermograms in Figure 10.

Phase Transition	Heating/Cooling Rate (K/min)		
	0.5	1.0	2.0
Low temp. eutectoid	58.0	52.8	17.6
High temp. eutectoid	120.3	119.7	119.8
Peritectic + Melting	28.2	28.5	27.7

**Table 7:** Summary of the influence of EG microparticles on the solid-solid transitions of pure NPG and TRIS: T_TR_ = solid-solid transition temperature at equilibrium; ΔH_TR_ = measured enthalpy of solid-solid transition on heating (referred to the total mass of the composite); ΔH_TR_ (cal.) = enthalpy of the solid-solid transition calculated by applying the rule of mixtures.

Compound	EG Content (%Wt.)	Undercooling (K)	T_TR_ (K)	ΔH_TR_ (Exp.) (J/g)	ΔH_TR_ (Calc.) (J/g)
NPG	0	14.3	312.6	119.4	119.4
	10	9.0	312.7	106.0	107.4
	20	9.5	312.5	95.0	95.5
	30	9.1	313.3	78.9	83.6
	50	10.7	313.4	53.0	59.7
TRIS	0	65.0	406.8	281.0	281.0
	5	55.4	406.1	230.8	267.0
	10	50.0	404.6	210.8	252.9
	15	47.0	401.1	199.2	238.8
	20	41.6	399.8	185.5	224.8

**Figure A1:**
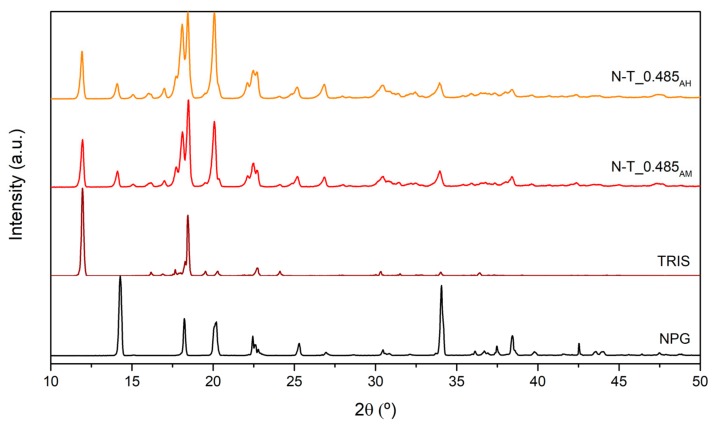
XRD diffractogram obtained at room temperature for (i) monoclinic structure of NPG (black line), (ii) orthorhombic structure of TRIS (brown line), (iii) NPG_0.515_TRIS_0.485_ after ball milling and mixing (red line) and (iv) NPG_0.515_TRIS_0.485_ after heating and cooling treatment (orange line).

**Figure A2:**
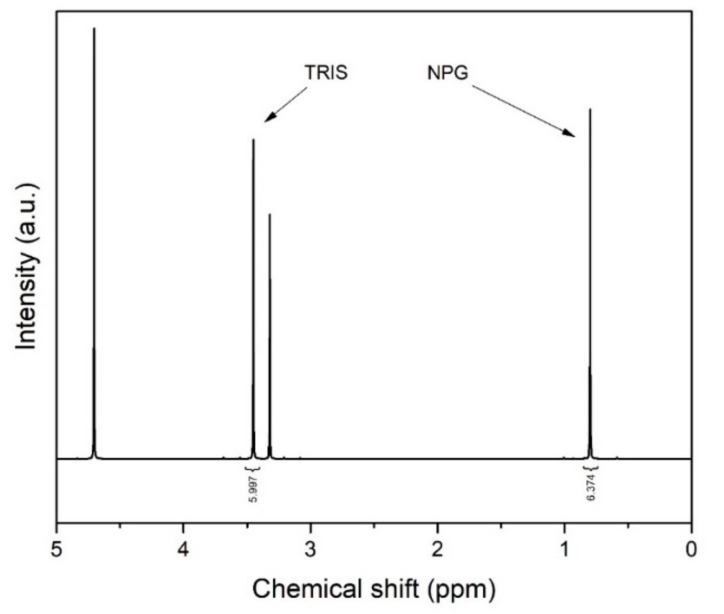
^1^H-NMR spectrum of the NPG-TRIS mixture with 0.485 molar fraction of TRIS.

## Appendix A

NPG_1−x_TRIS_x_ (0 < x <1) samples were submitted to several preliminary tests to ensure the pertinence of the results. XRD at room temperature was used to check that neither samples preparation nor their thermal treatment leads to degradation or structural changes compared to NPG and TRIS. An example of the results achieved is depicted in Figure A1. This figure shows the diffractograms of as received NPG (monoclinic structure) and TRIS (orthorhombic structure), as well as those of an NPG_0.515_TRIS_0.485_ sample just after milling/mixing and after melting and slow cooling down up to room temperature. The diffractograms of NPG and TRIS show the typical diffraction peaks of NPG monoclinic structure and TRIS orthorhombic structure, respectively. Regarding NPG_0.515_TRIS_0.485_, the diffraction pattern after milling/mixing is similar to that obtained after melting and solidification of the sample. It can be seen that the diffraction peaks of NPG and TRIS can be clearly recognized in the NPG_0.515_TRIS_0.485_ diffractograms as expected (see Figure 4). Moreover, none of them present new diffraction peaks. All this indicates that the crystalline structures of NPG and TRIS remain unchanged after milling/mixing and melting/solidification processes, proving that none of those processes induce degradation or new phases formation.

The composition of NPG_1-x_TRIS_x_ (0.45 ≤ x ≤ 0.55) was measured by liquid NMR before and after the heating treatment in order to verify that eventual NPG evaporation does not significantly change the initial composition of the sample. This technique was selected because both polyols (NPG, TRIS) are soluble in water and have distinct, nonoverlapping NMR spectra, thus leading to high accurate determination of their mixture composition. Indeed, as shown in Figure A2, the ratio between the number of methyl protons of NPG (chemical shift = 0.798 ppm) and methylene protons of TRIS (chemical shift = 3.450 ppm) can be easily determined. The signals at chemical shifts 3.338 ppm and 4.702 ppm correspond, respectively, to the methylene protons of NPG and water. The measurements of the composition of NPG-TRIS mixtures are reported in Table A1. It can be seen that there are no significant changes in the composition of the samples after heating treatment, thus concluding that evaporation of NPG is negligible under applied preparation and testing conditions.

**Table A1:** Composition (molar fraction of TRIS) in NPG-TRIS mixtures determined by ^1^H NMR.

**Before Thermal Treatment:**	0.45	0.46	0.47	0.485	0.485	0.485	0.49	0.5	0.51	0.52	0.53
**After Thermal Treatment:**	0.454	0.471	0.472	0.479	0.485	0.478	0.495	0.507	0.518	0.53	0.538
